# Overcoming phase 1 delays: the critical component of obstetric fistula prevention programs in resource-poor countries

**DOI:** 10.1186/1471-2393-12-68

**Published:** 2012-07-18

**Authors:** L Lewis Wall

**Affiliations:** 1 Department of Obstetrics & Gynecology, School of Medicine, Washington University in St. Louis, Campus Box 8064, 660 South Euclid Avenue, St. Louis, MO, 63110, USA; 2 Department of Anthropology, College of Arts and Sciences, Washington University in St. Louis, Campus Box 1114, One Brookings Drive, St. Louis, MO, 63130, USA

**Keywords:** Obstetric fistula prevention, Vesicovaginal fistula, Obstructed labor, Phases of delay, Trust

## Abstract

**Background:**

An obstetric fistula is a traumatic childbirth injury that occurs when labor is obstructed and delivery is delayed. Prolonged obstructed labor leads to the destruction of the tissues that normally separate the bladder from the vagina and creates a passageway (fistula) through which urine leaks continuously. Women with a fistula become social outcasts. Universal high-quality maternity care has eliminated the obstetric fistula in wealthy countries, but millions of women in resource-poor nations still experience prolonged labor and tens of thousands of new fistula sufferers are added to the millions of pre-existing cases each year. This article discusses fistula prevention in developing countries, focusing on the factors which delay treatment of prolonged labor.

**Discussion:**

Obstetric fistulas can be prevented through contraception, avoiding obstructed labor, or improving outcomes for women who develop obstructed labor. Contraception is of little use to women who are already pregnant and there is no reliable screening test to predict obstruction in advance of labor. Improving the outcome of obstructed labor depends on prompt diagnosis and timely intervention (usually by cesarean section). Because obstetric fistulas are caused by tissue compression, the time interval from obstruction to delivery is critical. This time interval is often extended by delays in deciding to seek care, delays in arriving at a hospital, and delays in accessing treatment after arrival. Communities can reasonably demand that governments and healthcare institutions improve the second (transportation) and third (treatment) phases of delay. Initial delays in seeking hospital care are caused by failure to recognize that labor is prolonged, confusion concerning what should be done (often the result of competing therapeutic pathways), lack of women’s agency, unfamiliarity with and fear of hospitals and the treatments they offer (especially surgery), and economic constraints on access to care.

**Summary:**

Women in resource-poor countries will use institutional obstetric care when the services provided are valued more than the competing choices offered by a pluralistic medical system. The key to obstetric fistula prevention is competent obstetrical care delivered respectfully, promptly, and at affordable cost. The utilization of these services is driven largely by trust.

## Background

Labor becomes obstructed when the progress of the fetus through the pelvis is arrested, in spite of ongoing, vigorous uterine contractions [1,2]. Untreated prolonged obstructed labor is one of the five most common causes of maternal mortality in poor countries (the others being hemorrhage, hypertensive disorders of pregnancy, sepsis, and complications of unsafe abortion) [2]. In addition to being a major cause of maternal death, obstructed labor also creates obstetric fistulas, one of the most devastating kinds of severe obstetric morbidity [3]. An obstetric fistula is formed when the tissues that normally separate the vagina from the bladder and/or rectum are destroyed by the prolonged impaction of the presenting fetal part (usually the fetal head) against the soft maternal tissues that are trapped between the fetal head and the woman’s boney pelvis. The destruction of these tissue barriers joins these adjacent structures through a fistula, which results in continuous incontinence of urine or stool (sometimes both) and turns the affected woman into a social outcast. The misery experienced by a woman with an obstetric fistula is incalculable [4,5]. Estimates suggest that as many as 3.5 million women currently suffer from this condition in developing countries (primarily in sub-Saharan Africa and south Asia), with an annual incidence of between 50,000 and 130,000 new cases per year [3,6].

A fistula forms when obstructed labor puts enough pressure on the soft maternal tissues trapped between the fetus and the woman’s pelvic bones to compromise their blood supply. As blood flow is cut off, the tissues eventually cross a threshold at which tissue death occurs. This threshold is affected by many different factors, including the amount of force acting on the tissues, the location at which obstruction occurs, the length of time labor has been obstructed, and the inherent resilience of the affected tissues (itself a complex summation of many interconnected biological factors). Because a complicated interplay of factors sets the threshold at which injury occurs, there is no obvious minimum time limit after which an obstetric fistula will be produced. Relatively short labors less than 12 hours in length may result in a fistula if the conditions for “a perfect storm” are present [7]. In practical terms, this means that all cases of obstructed labor should be regarded as emergencies and treated promptly to avoid the development of serious complications. As Thomas Addis Emmet recognized nearly 150 years ago, “…the amount of injury is by no means in proportion to the length of labor. Therefore, the only safety consists in as speedy a delivery as the circumstances of the case will admit” [8], (p. 20). In addition to the formation of obstetric fistulas, a wide array of other pelvic injuries--- commonly referred to as “the obstructed labor injury complex” [9]---may accompany obstructed labor.

## Discussion

### Reducing maternal mortality and severe obstetric morbidity

It has been obvious for many years that reductions in maternal mortality and serious maternal morbidity can only be accomplished through three mechanisms [10]:

1.The risk of becoming pregnant can be reduced. Since only women who become pregnant are at risk for maternal death or severe maternal morbidity, family planning programs can reduce the number of pregnancies in a given population and thus reduce the number of women at risk for pregnancy complications [11,12].

2. The risk of a woman developing a complication during pregnancy might be reduced. This is the rationale for antenatal screening programs. Unfortunately, the risk-approach to reducing maternal mortality and morbidity has not worked in practice because the vast majority of life-threatening obstetric complications arise unexpectedly and cannot be predicted in advance [13-15]. In spite of decades of effort using both high- and low-technology approaches, there are still no screening tools with adequate sensitivity and specificity for routine clinical use that will predict which women will develop obstructed labor in advance of labor itself [16-20]. The most reliable predictor of obstetric outcome is past obstetric history, a fact that is particularly unhelpful for evaluating potential complications during a woman’s first pregnancy [21].

3. Outcomes can be improved for women who develop complications during pregnancy, labor, and delivery. Obstetric emergencies such as hemorrhage, eclampsia, sepsis, or obstructed labor require prompt treatment if death or serious morbidity is to be avoided. Improving the outcome for women who develop complications is the rationale for enhancing rapid access to emergency obstetric services. This has emerged as the cornerstone of current efforts to reduce maternal mortality and morbidity in developing nations [22,23].

The treatment that is most likely to improve both maternal and fetal outcomes in obstructed labor is cesarean section, which by-passes the obstruction in the birth canal by creating an abdominal alternative to vaginal delivery. For injury to be avoided, delivery must take place expeditiously, with minimal delay. The longer labor remains obstructed, the longer potentially damaging forces are applied to the vulnerable soft tissues of the affected woman’s pelvis, and the more likely it is that pressure necrosis will produce an obstetric fistula. Therefore, the key to obstetric fistula prevention is prompt diagnosis and treatment of obstructed labor.

### The three phases of delay in obstetric emergencies

In their groundbreaking article “Too far to walk: Maternal mortality in context,” Thaddeus and Maine proposed a three part framework for evaluating delays in accessing emergency obstetric care [24]. This framework is based on the understanding that most life-threatening emergencies cannot be predicted in advance and that once a complication arises, a bad outcome can only be avoided by the prompt provision of effective treatment. In any emergency scenario, a similar series of steps occurs: development of a complication, recognition that a complication has arisen, a decision to seek treatment for the complication, movement to a center where emergency obstetric services are provided, and delivery of care that resolves the complication. Not all cases of maternal death or serious maternal injury can be prevented, but if the time between the onset of the complication and the delivery of appropriate treatment is minimized, overall outcomes will be dramatically better. This is particularly true in cases of obstructed labor, where trauma to maternal tissues sufficient to produce an obstetric fistula is likely to take at least several hours before the injury threshold is crossed. Of all of the major complications that a woman could develop during labor, the formation of an obstetric fistula should be the most preventable.

Thaddeus and Maine articulated three principal “phases of delay” impacting the successful resolution of life-threatening obstetric emergencies: 1) delay in deciding to seek care; 2) delay in arriving at a suitable obstetric care facility; and 3) delay in receiving appropriate care at that facility [24]. The three phases of delay are each influenced by different factors and the solutions to the impediments that cause delays in each phase can be provided by different actors. In this scheme of things, phase two and phase three delays appear to be the most amenable to solution.

There is abundant evidence that maternal death and serious birth injury increase with increasing distance from medical facilities [22,25-28]. Most travel delays (phase two delays) could probably be overcome by a combination of community mobilization and the establishment of emergency obstetric transportation networks (ambulances, etc) linked by an effective communications system that ties the various components of the healthcare system together [29-31]. This is the kind of project that people can reasonably demand from their governments and healthcare systems. Doing this is an essential part of public service. For example, in the 1990s when the government of Honduras set about trying to reduce the high maternal mortality that existed in the Department of Intibuca, an inaccessible mountainous region in the west of the country along the border with El Salvador, they constructed hospitals and health centers, linked them with an ambulance service, created a radio communications network to improve efficiency, and in the case of the town of San Francisco de Opalaca (which had the highest maternal mortality in the country and which was inaccessible except by foot), they pushed a new road through to open the community to outside access [32]. Sustained efforts to develop infrastructure in this way have also played a major role in improving maternal health in countries such as Sri Lanka and Malaysia [33].

The problem of phase three delays (delays in the delivery of competent emergency care) properly falls within the purview of public health officials, hospital administrators, physicians, midwives, and their professional organizations. Deaths and injuries that occur after a woman has arrived at a hospital are often due to incompetent decisions, neglect of patients, shortages of supplies and personnel, or other logistical factors [34-37]. It is not unreasonable to demand accountability within the medical system for such shortcomings and to demand that such problems be solved. Instituting tight administration, vigilant oversight, protocols for the treatment of common problems such as obstructed labor, and ongoing professional development and self criticism will go a long way towards resolving phase three delays. People should rightly demand timely deliverance of competent medical care from the hospitals and health centers that serve them [38-42].

### Overcoming phase I delays: the critical component of obstetric fistula prevention

In both phase two and phase three delays, it is at least theoretically possible to allocate responsibility for poor system performance and to demand accountability for the high rates of maternal death and disability that result. This fact offers at least the possibility of leveraging improved performance for the public good. This is largely a political problem, but one that can be overcome even in low-resource countries [43,44]. The more complicated problem appears to be changing the causes of phase one delays, because these depend largely upon individual behavior. For this reason we refer to phase one delays as “the critical component in obstetric fistula prevention.”

If the process of accessing emergency care outlined by Thaddeus and Maine is to function effectively in the first phase, several things must happen in rapid sequence: a problem must be present, it must be recognized as a problem important enough to require action, the action needed to solve the problem must be identified and agreed upon (usually invoking some conception of cause and effect or at least identifying the “repository of knowledge” where the solution to the problem can be found), and a decision to act must be made that moves the process into the second and third phases of care-seeking. The factors that impact attitudes and decisions in this critical first phase are far more diffuse, intangible, and difficult to control than those impacting delays in the second and third phases because these factors operate at the level of individual and family dynamics. As Edward de Bono has written, “Most of the mistakes in thinking are inadequacies of perception rather than mistakes in logic” [45], (p. 58). In the first phase of delay, perception is everything. Understanding that a problem is present, understanding what the problem is, and understanding how the problem may be solved are absolutely critical for the successful resolution of obstetric emergencies.

Obstetric fistula has been eliminated in wealthy countries where educational standards are good and prompt access to emergency obstetric care is the cultural norm. If a woman in an affluent country develops a serious injury from obstructed labor, the event is remarkable enough to be written up and published as a case report in a medical journal [46,47]. In contrast, there are millions of unrepaired obstetric fistulas in sub-Saharan Africa and south Asia. The epidemiology of obstetric fistula clearly indicates that women who develop this condition come predominantly from poor communities, usually located in the rural areas of resource-poor nations, where women have limited access to formal education, marry early (often while still children themselves), have high rates of fertility, and usually lack employment opportunities that would generate a significant cash income and lead to greater personal autonomy [7,48-52]. For example, among 899 women who developed an obstetric fistula and presented for care at Evangel Hospital in Jos, Nigeria, the mean age at marriage was 15.5 years, most were illiterate, 78% had no formal education, only 4.5% had ever used contraception, and only 10 women had any kind of regular paid or salaried employment. The rest were all housewives, agricultural workers, menial laborers, or earned what little income they had through petty trading [48].

Because fistula patients tend to be poor, lacking in formal education, and immersed in rural African or Asian culture, there are deep social components to the obstetric fistula problem which have not yet been adequately explored by researchers. Although all three phases of delay are influenced by cultural factors, these influences appear to be most pronounced in the first phase of delay during which the existence of a problem is perceived, the possible solutions to the problem are debated, and a decision is made to seek a solution to the problem as perceived. Perhaps the most fundamental point to be made is that the decision to seek therapy is not simply a decision either “to do nothing” or to “go to a hospital.” Women with pregnancy complications in rural African and Asian communities can utilize many competing therapeutic options, albeit of greatly differing therapeutic efficacy, especially when it comes to obstructed labor. Therapeutic pluralism is the norm in these communities and the quest for therapy often involves the concurrent use of multiple different healing pathways depending on how the presenting problem is understood and how the efficacy of competing therapies is evaluated [53,54]. From the standpoint of bioscientific obstetrics, obstructed labor is a problem of faulty obstetrical mechanics---the fetus will not fit through the birth canal---but those most intimately affected by a case of obstructed labor may be more worried about social factors than about simple mechanics. Their concerns may lie more with metaphysics than with physics proper. They may be more concerned with placating the supernatural forces they fear may be responsible for the delay in delivery than with understanding and rectifying the faulty obsetrical mechanics involved. Such concerns have significant implications for what happens next.

### How long should a normal labor last?

The formation of an obstetric fistula is a problem that originates during prolonged labor when that labor is obstructed. The critical problem in the first phase of delay is recognizing that labor is prolonged. By WHO standards, labor is prolonged if it lasts more than 24 hours [55] and there is an old adage in tropical obstetrics that “the sun should not rise twice on a laboring woman.” If a woman is laboring under the supervision of a skilled birth attendant, the diagnosis of prolonged/obstructed labor should not be difficult to make, but in parts of the world where fistulas are common, most women deliver by themselves, in the company of family members, or using other forms of traditional birth assistance [56,57]. Under these circumstances, what is regarded as the “normal” length of labor may be quite different from accepted obstetrical norms. For example, The Prevention of Maternal Mortality Network in West Africa found that in Bo, Sierra Leone, and in the Nigerian cities of Sokoto and Zaria, prolonged labor was not considered a problem of sufficient importance to seek medical care until two to five days had elapsed [58]. As Douglas and Wildavsky have pointed out, “risk” is constructed differently in every culture, based on local perceptions and values, and “substantial disagreement remains over what is risky, how risky it is, and what to do about it” [59], (p 1). The concepts of “risk” and “blame” are deeply anchored in local cultural constructs and underlying assumptions about the nature of the world [60,61]. Determining when labor is actually prolonged depends very much upon local notions of what the “normal” length of labor might be [54]. Anthropologists are only just beginning to investigate cross-cultural notions of time in relation to pregnancy, labor and delivery—concepts which are critical to obstetric fistula prevention [62]. Added to the problem of determining the “normal” length of labor is the difficulty of distinguishing “false” labor (irregular uterine contractions that may mimick labor but which do not produce cervical change) from “true” labor (uterine contractions of sufficient force, duration, and frequency to produce effacement and dilatation of the cervix)—a diagnosis that is difficult to make without performing serial cervical examinations [63]. Distinguishing labor that is prolonged due to ineffective uterine contractions from labor that is truly obstructed, also takes obstetrical experience. Tracking the progress of labor with a partograph (a simple graphic depiction of the progress of labor) is extremely effective in determining when labor is prolonged [64,65]. Intensive and ongoing community education programs that emphasize the importance of seeking skilled care if labor lasts more than 24 hours are likely to be fundamental in altering traditional attitudes about the “normal” length of labor.

### What is to be done?

Once it has been determined that labor is prolonged, a solution to this problem must be proposed. For effective fistula prevention, the laboring woman should be transported rapidly to an emergency obstetric care facility where proper treatment (often cesarean section) can be provided. At this point, however, there are many possible and very divergent therapeutic pathways that can be chosen by the actors involved. Some of these pathways—perhaps many---will lead to adverse outcomes including death or severe morbidity when labor is obstructed.

One decision is simply to do nothing. In clinical medicine this is referred to as “watchful waiting,” and while avoiding *unnecessary* intervention is often a virtue in obstetric practice [66], this approach can be disastrous if it allows labor to drag on for several days. In some cases the decision to do nothing is based on religious fatalism—if God so wills, the problem will resolve itself. In other cases the parties involved may simply have no idea of what to do or where to turn, and so do nothing. In the study of 899 fistula cases from Jos, Nigeria, by Wall and colleagues, 6.5% of patients reported that they were unaware of the availability of hospital obstetric care and nearly 27% could not give any reason as to why they delayed seeking help [48].

In some cases an intervention *other* than transporting the patient to an obstetric emergency care facility will be chosen. One common therapeutic option is to seek help from someone local who is regarded as an authority on problems associated with childbirth. Such individuals function as repositories of “authoritative knowledge” with respect to childbearing difficulties, “the source” in which solutions can be found within the local cosmology [67]. These authorities may be midwives or shamans or religious figures (pastors, priests, imams, etc) who are thought to possess special skills or information that may be of therapeutic utility in difficult cases of labor. In Christian Africa, churches are often the first place of refuge in cases of dystocia. Therapy typically consists of prayer and religious rituals that do not effectively address the problem of mechanical obstruction [68,69]. Muslims often resort to versions of Islamic folk medicine, attempting to harness the power they believe resides in the Koran. In northern Nigeria, for example, a common treatment for many ills is writing out on a wooden slate a verse from the Koran that is thought to be “therapeutically potent,” washing off the ink that has been used to write (and therefore embody the power of) God’s words, thereafter drinking the inky water as a medicine [70]. In other parts of Africa traditional lineage priests or clan elders may be convened to discuss the case, particularly if there is suspicion that the pregnant woman has committed adultery or other sins which are blocking her delivery [58,71-74]. The Prevention of Maternal Mortality Network found “In all of the areas studied, certain behavior (including infidelity and disregarding the authority of one’s husband or elders) is believed to lead to obstructed labor and hemorrhage. Women in Accra, Benin, Bo, Calabar, and Freetown reported that when complications arise, the oracles are consulted, and if, for example, the oracle says the complication is due to the woman’s insubordination to her husband or elders, she has to apologize and perform cleansing rites before she is taken for treatment. Similarly, in Bo, a woman suffering from a complication thought to be due to infidelity is forced to confess her sins, and her husband must spit water on her abdomen to appease the gods; only then is she taken for further help, if the complication is thought serious enough to warrant hospital treatment. In most of the communities studied, people believe that the will of God, heredity, and evil spirits can cause obstetric complications. In such situations, the care of traditional healers and diviners is sought, and the modern health-care system is used only as a last resort” [58].

Beliefs that problems in labor arise from disturbances in the social environment rather than as simple problems of obstetrical mechanics are common in many cultures, and women are often blamed for these, and other, health misfortunes. Among the Esan people of Edo State, Nigeria, “It was observed from discussions with both men and women that illnesses in adult women are mostly caused by offenses against tradition or custom. In contrast, the illnesses of adult males and children are seldom self-inflicted but are often caused by the misdeeds of women. In essence, a woman is blamed for disasters to her child, her co-wives’ children, and her husband; but she alone must bear the responsibility for her own state of health” [71]. Obstructed labor—recognized as a condition potentially fatal to both mother and child—is thought to be caused by factors such as “having sex in the afternoon or in the fields, incest, adultery, practicing witchcraft and taking a husband’s property (such as money) without his knowledge or permission. Most of these supernatural factors can be brought promptly under control when the woman confesses her offence, which is necessary before ritual can be successful; otherwise, no cure can be provided and death becomes inevitable” [71].

According to Monica Wilson, the Nyakyusa of East Africa believe that “A delayed delivery is commonly attributed to the woman’s adultery, and she is pressed by the midwife to confess the name of her lover or lovers, but it is also believed that it may be due to *imindu*, that is the shades [ancestral spirits]. The husband consults a diviner who indicates whether the *imindu* is on his side or that of the woman’s father and the one who is thus indicated should pray. ‘Sometimes the woman herself tells of a quarrel which would lead to *imindu* and then her husband or father goes to pray’” [72] (p. 144).

Denise Allen described a belief called *usangalija* among the Sukuma of west central Tanzania. *Usangalija* is the Sukuma term for prolonged or stalled labor. It refers to the phenomenon of “mixing,” when a woman allows “foreign” sperm to enter her while pregnant with a baby fathered by a different man. This “mixing of men” is potentially dangerous for both the mother and child, for the outcome of such cases may be fatal. It is said to produce a sort of revulsion on the part of the fetus, who “instead of moving down the birth canal, … moves up in uterus instead” [73] (p.205). Allen recounted several stories of women who were accused of adultery after they experienced difficulties during childbirth. The proposed local treatments of *usangalija* included such therapies as taking a pinch of sand from the exact spot where a dog—a notoriously promiscuous animal species---had previously given birth, mixing it with water and giving it to the laboring woman to drink, or taking a root found growing in the middle of the road---a place through which much traffic has passed---grinding it, mixing it with water, and giving to the woman to drink.

In other cultures sexual misbehavior on the part of the husband is also thought to affect a pregnancy. According to Chapman, among the Shona of Mozambique, “Adultery on the part of the husband can also kill his pregnant wife: if the woman with whom he has had the affair comes near the wife while she is in labor, the wife will begin to sweat and then die.” [74] (p.125). More commonly, however, the problem is attributed to infidelity on the part of the pregnant woman. This has profound implications for family dynamics. As Chapman writes, “In a patrilocal marriage, where a wife moves to live with her husband and his patrikin, a mother-in-law *sogra* can also exercise considerable influence over her son’s wife if she experiences trouble with childbirth. Infidelity on the part of the pregnant woman is widely believed to cause problems during childbirth, especially prolonged or blocked labor, and it is the right and duty of a mother-in-law to extract this information from her daughter-in-law. Armed with such a confession, the *sogra* can inform her son, often initiating a break in relations between the young couple or even catalyzing divorce proceedings, thus fortifying her own position of influence with her son. Senior women’s power in this setting is linked to their ritual control over certain diagnoses in pregnancy and birthing that carry social meaning” [74] (p.215).

It is critical to understand that for women in many cultures, obstetric problems such as prolonged labor are not viewed as random physiological events but rather are tied directly to their unique individual relationships with their family and community. Anthropologist Nicole Berry recounted the following explanation from one of her Mayan informants in rural Guatemala: “Sandra told me that one of her births had taken more than five days. Why did it take so long, I asked? Probably, she said, because she had been fighting a lot with her sisters-in-law during the pregnancy. As the birth is a family event, if things are not going well within the family, they might not go well within the birth. A bad relationship between a husband and wife, the central actors in the birth narrative, can be the root of even worse problems. Husbands who don’t take care of their wives and fight a lot with their wives while they are pregnant were also blamed for causing birthing problems” [75] (p.171).

When traditional midwives are consulted for obstetrical problems, they follow their own culturally-derived diagnostic and treatment logic, which is usually quite different from that advanced by biomedical obstetrics [54,73,76,77]. This may lead to therapeutic decisions that seem logical within the local context but which are ineffective or even directly harmful to the laboring woman. For example, it may be decided that the uterus is not contracting strongly enough. To combat this, an oxytocic drug may be administered, either in the form of a traditional recipe using locally obtained bioactive materials [78] or in the form of a standard pharmaceutical preparation obtained on the black market or elsewhere [79,80]. In obstructed labor this will usually increase the force applied to the impacted fetal part, thereby increasing the likelihood of uterine rupture or fistula formation. In some cases violent external force—such as sitting on the pregnant woman’s abdomen—may be applied to try to force the baby out, with disastrous consequences [72] (p.181).

In other cases crude attempts may be made to release “the obstruction” by cutting inside the vagina. The traditional ethnomedical system of the Hausa people of northern Nigeria, for example, recognizes the existence of a condition called *gishiri*. *Gishiri* is the Hausa word for “salt,” and when used as a medical diagnosis it refers to a condition believed to occur when dietary imbalances in the intake of salty and sweet substances cause a web or membrane to grow over the vagina, resulting in obstructed labor. (The term *gishiri* is also used to refer to the salts that encrust the bottoms of water-pots as the water leaches through the clay and evaporates on the outside—a process of encrustation seen as analogous to the pathophysiological process that obstructs the birth canal). This condition is treated with surgery: a barber or midwife takes a razor, knife, or other sharp object and make a series of cuts inside the vagina, often filleting the urethra, in an attempt to remove the obstruction. This practice itself frequently creates a fistula through direct urethral or bladder injury [48,70,81,82].

### Who decides what…and why?

Because traditional ethnomedical therapies have little efficacy in relieving obstructed labor, the most critical decision in the prevention of obstetric fistulas is the decision to seek help from a biomedical facility that provides competent emergency obstetrical services, including cesarean delivery. It is the decision to seek help in such a venue that starts the laboring woman down the therapeutic pathway that may save her life as well as prevent the development of a fistula. Who decides this? In many cases the woman herself may have little or no say in this critical decision.

In many societies where obstetric fistulas are common, women often have little independent agency [83-85]. They may have little choice as to when they have sexual relations and whether or not to use contraception when they do [86,87]. Contraceptive agency is affected strongly by male attitudes [88], but also by social factors unknown in the West such as the presence of co-wives in polygamous households [89]. When women in fistula-prevalent areas become pregnant, they may have little say as to whether or not they get antenatal care---and where and under what circumstances—they deliver their children. In many societies “proper” social relationships require that female reproductive capacity is always under clearly delineated male control, usually by the girl’s father before marriage and by her husband after marriage. Money and material goods (“bridewealth,” in anthropological parlance) [90,91] are transferred by the husband and his family to the girl’s family as part of the marriage contract in exchange for the use of her reproductive capacity and the assumption of other obligations on their part. The rights and obligations entailed by such practices vary enormously from society to society, but if males believe that they in some sense “own” a woman’s reproductive capacity, this may significantly impact decision-making during obstetrical emergencies. Among the Hausa of northern Nigeria, for example, children are referred to as “the profit” (*riba*) from the marriage transaction [70,92]. Analogies of children being the “harvest” obtained by a man as the result of “tilling” his wife’s “field” are explicit agricultural analogies in many countries [93]. The control of such a valuable resource is not easily relinquished, and if wife seclusion (*purdah*) is the prevailing cultural practice (as it is among the Hausa), a woman may not be allowed to leave her family compound without explicit permission from a controlling male authority [70,92-94]. The consequences can be devastating. There is a famous anecdote concerning a woman from northern Nigeria who lived a 10 minute walk from the hospital but because her husband was away on business and could not give her permission to travel, she labored at home for several days only to deliver a dead child and develop a fistula [58]. Wife seclusion also greatly limits female economic opportunity, further reducing women’s agency and making them financially dependent on their husbands [95-99]. Lack of formal education further increases this sense of helplessness in the face of obstetrical complications [100,101]. Fistula patients almost invariably have low educational attainments, as noted previously [48].

Weeks and colleagues interviewed 30 Ugandan women who had ‘near miss’ obstetrical experiences at Mulago Hospital that might have proved fatal if circumstances had been different. In analyzing the recurrent themes in their interviews, they noted “The most striking feature is the women’s descriptions of their powerlessness, which was seen in all aspects of their lives” [102]. The authors explained: “Traditionally in Ugandan culture, the roles of men and women are strictly defined with men being breadwinners and the women homemakers. Their background of poverty and limited education restricts their ability to control their own lives. For many families, this places women in a subservient role within relationships, relying heavily on their male partner for financial support and decision-making, and being sexually compliant and looking after the home and family in return. A dysfunctional form of this arrangement was seen in many interviews, with women left hungry, ignorant, or even raped” [102].

### The power of fear in promoting delay

Fear of the biomedical healthcare system is also a potent factor which delays the decision to seek help when labor is obstructed. There are many different facets to this fear: fear of the unknown, fear of ridicule and abuse by the hospital staff, fear or receiving poor quality care, and fear of being forced to undergo an unwanted---and perhaps unnecessary--- surgical operation—all fears which may be justifiable, depending on the locale and the context. For women (and their families) who live in rural areas with little formal education and limited interactions with more cosmopolitan communities, the prospect of going to a biomedical health facility may be daunting.

In many cultures where a woman’s status is determined primarily by her reproductive capacities, the ability to deliver a baby “on her own” is important in validating her status as a fully adult woman. Failure to deliver vaginally may be stigmatized as a form of reproductive failure. The fact that cesarean delivery may be life-saving is not always widely understood. In a study of the use of maternity services in Uganda, Grace Kyomuhendo reported that Ugandan women regarded pregnancy and childbirth as a journey “on a thorn-strewn path,” the successful traversal of which entitles a woman to be praised as *garukayo* (“dare to go back”) [103]. She writes, “The traditional praise *garukayo* far supercedes mere praise of the new mother, but is also meant to remind her that the hardships experienced notwithstanding, she has no option but to prepare to get pregnant again. … The way a woman endures pregnancy and birth therefore has implications for her position in her household and community. One who experiences no problems and needs no assistance is held in much esteem, having walked bravely and emerged unscathed. One who experiences a difficult pregnancy, perhaps requiring hospitalization, an episiotomy or caesarean section, is not respected and is referred to as *omugara* (lazy), though the circumstances are beyond her control. To seek external help is to stumble and such women even after delivery do not deserve a genuine *garukayo*” [103] Attitudes of this kind are very common in parts of the world where obstetric fistulas are prevalent [54,104].

Aside from the belief that cesarean delivery is somehow “unnatural” and therefore represents a failure at the most elemental level of womanhood, a cesarean section is also a major abdominal operation that inevitably causes pain and may be accompanied by complications, particularly when the surgery is performed for difficult cases of obstructed labor in low-resource settings by surgeons who may not have top-notch obstetrical skills [105-107]. The combination of unfavorable attitudes towards cesarean section [108-112] and the frequent need for repeat cesarean delivery in subsequent pregnancies due to recurrent dystocia and the risk that the uterine scar may rupture during labor, means that many women have already been told that if they have the operation they will need a repeat cesarean later. Dissatisfaction with earlier experiences may contribute to delay until catastrophe strikes.

Fear may be compounded by linguistic confusion. As Nicole Berry points out in her study of maternal health in Guatemala, “Operations have no parallels in ‘traditional’ medicine that Kaqchikel villagers used, and on an intuitive level it is not difficult to understand why they are so unpopular. Cutting a body open seems inherently invasive and dangerous, and it is difficult to imagine that anyone weak, sick, or compromised could have the strength to survive such an ordeal.” [75] (p. 174). Blood is often needed and frequently is not available. Constant requests to donate blood for operations make villagers leery of being exploited by having a valuable resource extracted from their bodies by powerful government officials and the word “*operacion*” was frequently used to refer both to cesarean delivery as well as to tubal ligation, which would end a woman’s chances of having further children. The result of this situation was that “Women and their families feared that if they went to the public hospital for a c-section, they might come out unable to have more babies,” [75] (p. 182).

The conditions under which care is provided and the attitudes of hospital staff towards patients may create a situation in which going to the hospital or health center is seen as a decision of “last resort.” Many healthcare facilities in low-resource countries are understaffed, poorly supplied, and overwhelmed with patients, thereby producing highly stressful conditions in which, even with the best of intentions, adequate care cannot be provided [110-121]. Abuse and neglect of patients by under-skilled and overworked doctors and nurses is commonplace in such circumstances. One study in Gabon documented a much higher case fatality rate among women seeking care for abortion complications compared to other obstetric emergencies, a fact that was linked to a delay between diagnosis and treatment that was 20 times longer for abortion complications than for post-partum hemorrhage or eclampsia. The delay in treatment and the high case fatality rate was attributed by the authors to cultural stigmatization of patients by health care personnel. In these cases, disdain for patients turned out to be fatal [119]. In China, where the government has adopted a rigorous policy of limiting family size, women who become pregnant “outside permitted limits” (perhaps in quest of a son), often avoid institutional maternity care so that they will not be abused, harassed, stigmatized, or punished for their pregnancies, sometimes with fatal results [122].

### Economic constraints on the decision to seek care

Even when the problem is clear, when the location at which help may be obtained has been identified, and when the fears surrounding possible treatments (such as cesarean section) have been overcome, there may still be substantial economic barriers that delay or prevent access to necessary care. The economic costs of obtaining medical care at a hospital or clinic derive from many sources and the sum of these costs may be beyond the budget of all but the most affluent families [123-133]. Particularly in remote areas, the costs of transportation required to reach a hospital may be substantial, sometimes more than the cost of care itself [124]. There are costs not only for the patient herself, but also for accompanying family members. There are food costs for the patient and her family members both while traveling and during the period of hospitalization. There are opportunity costs that result from going to the hospital rather than selling goods in the market, working in the fields, or engaging in other forms of economic activity. In Tanzania, Kowalewski has reported that women over age 35 and women with more than four children actively avoid hospital delivery because they need to provide farm labor and child care and nobody else is free to provide these necessary services [124]. For people in subsistence or marginal economic circumstances, such opportunity costs may be an insurmountable barrier.

Emergency obstetric care often involves both “formal” charges from the healthcare system, as well as “unofficial” (but still very real) costs incurred for necessary goods and services. In many cases healthcare institutions have instituted user fees to help recoup the costs of providing services, but such fees disproportionately affect women, children, and the poor, with adverse health consequences [134-137]. Such charges also diminish the utilization of services, adversely affecting the most vulnerable population groups. But even if care is ostensibly “free,” the patient and her family may still incur substantial “informal” charges---costs of supplies and medications as well as bribes and gratuities for access and services—that can dwarf other expenditures [138]. As Afsana wrote of her research on obstetric costs in Bangladesh, “When emergency surgical procedures such as caesarean sections were required, the urgency put poor villagers under tremendous stress to secure the money. Families would arrive at the hospital with some cash, but the amount of money required was beyond their imagination.” [133]. Furthermore, “Collecting the required money was difficult for poor villagers, who usually had no assets or savings. No one wanted to loan them money either. Some families borrowed money from moneylenders at very high interest rates, which tripled within six months. Some raised money by selling domestic birds, cattle or land or even a tin shed roof” [133].

These combined costs—opportunity costs, formal charges, and informal payments—often reach catastrophic levels which may consume over half of a family’s annual income [125,126,128,130,133]. Nahar and Costello found that over 20% of families were spending between 51% and 100% of their monthly income to pay for a “free” delivery in Bangladesh and that 27% of families were forced to spend between 2 to 8 times their monthly income to cover the costs of complicated maternity care [128]. A Pakistani study on obstetric costs found that both vaginal delivery and cesarean section were beyond the limits of what three-quarters of Pakistani households could afford [139]. Similar results have been found in Ghana and Benin, leading the authors to conclude that “For those women who require hospital delivery, accessing sufficient cash to cover the bill can cause significant delays in receiving treatment” [126]. Among the poorest of the poor, the need to finance expenditures of such magnitude may result in permanent financial struggles and submersion in a cycle of debt and impoverishment from which they cannot escape [140]. After investigating the costs of emergency obstetric care in Burkina Faso, Storeng and colleagues wrote: “A pervasive theme in in-depth interviews was anxiety about the costs of care. … A caesarean section, which in Burkina Faso is performed almost exclusively as a life-saving intervention, was widely held to presage unaffordable costs, potentially accompanied by social calamity if it meant that a woman was divorced or abandoned on account of being ‘too expensive’” [130]. The result of these economic factors is that large segments of the population in the world’s poorest countries have almost no access to cesarean section and it is among these women that the obstetric fistula problem is most pressing [141]. While many women are *willing* to pay to receive life-saving care, even under the best of circumstances they may simply not be *able* to pay [142]. The consequences of this economic situation are tragic. The terse observation that “inequities in maternal mortality are largely shaped by social, economic and political vulnerabilities that disproportionately affect the world’s poor” is quite accurate [130].

## Summary

### The importance of trust

Women in prolonged or obstructed labor will use scientific obstetric care when they and their families value the services provided by healthcare institutions more than they value the competing choices offered by a pluralistic medical system. For biomedical obstetric services to be valued, the community must understand that obstructed labor is a physical impasse that develops from abnormal obstetrical mechanics which can be reliably corrected by an appropriate mechanical intervention—if that intervention occurs in a timely fashion. The community must also understand that the consequences of not intervening quickly during obstructed labor can be devastating and deadly. The care that patients receive must be perceived to be both effective and of high quality. Care must be socially as well as physically accessible and it must be regarded as worth the social and economic costs involved. Those costs must also be affordable within the local socio-economic context. This does not necessarily mean that care must be free. As Kruk and colleagues noted in their study of maternity services in rural Tanzania, “The fact that at least some women in this population were willing to pay more than twice as much to deliver in mission facilities rather than government facilities underlines the importance of quality of care to women in rural areas” [124].

None of these conclusions is particularly striking by itself. What is striking are the ways in which all three phases of delay are linked together. Delays in obtaining effective emergency care depend in large part upon the feasibility of getting to an appropriate healthcare facility in a timely fashion (Phase 2 delay), but the decision to set out on such a journey is tied directly to the perceived quality of the care that will be obtained at the final destination (Phase 3 delays). At the most fundamental level all healthcare systems are driven by trust [143-145].

Phase 1 delays occur when the decision to seek effective care is postponed. There is abundant evidence that emergency obstetric care is sought more quickly and is rendered more effectively to women who are registered in an antenatal care system. Prenatal care plays a crucial role in obstetric fistula prevention, not because those women who will develop obstructed labor can be predicted accurately in advance, but because women who are already “booked” in the system are more likely to get emergency care promptly than are unregistered women. Kelsey Harrison’s magisterial study of nearly 23,000 hospital births in Zaria, northern Nigeria (a work which almost single-handedly launched the Safe Motherhood Initiative), demonstrated the dramatic effects of antenatal care and formal education in improving maternal and child health in resource-poor settings [146]. Among illiterate, unbooked women (33% of his study population), the maternal mortality ratio was a stunning 2,900 maternal deaths per 100,000 deliveries with a perinatal mortality of 26%. Among educated women registered for antenatal care (10% of the study population), the maternal mortality ratio was over ten times lower (250 deaths per 100,000 deliveries) with a similar reduction in perinatal mortality (only 3%). Multiple studies of uterine rupture (an often-fatal end result of prolonged obstructed labor) show similar outcomes: delay and disaster are far more common among unbooked pregnancies [147-151]. Taken together, the data indicate that competent obstetrical care which is delivered respectfully, promptly, and at affordable cost is the key to obstetric fistula prevention, but such care will not be effective unless it is utilized by the women who need it.

Overcoming the cultural barriers to the utilization of care requires a multifaceted approach. Intensive community education about obstructed labor combined with an efficient, welcoming system of prenatal care and competent, accessible emergency obstetric services is fundamental to reducing the burden of obstetric fistulas. Particularly in the rural communities where fistulas are prevalent, it is critical that men understand the vital stake that they themselves have in the health of their mothers, wives, sisters, and daughters. Men can play an extremely important role in making the system of maternity care function effectively in such settings. Thoughtfully structured programs that increase the availability of skilled midwifery care at the local level can be particularly effective in raising awareness of these issues, especially if such programs are combined with a vigorous, ongoing social marketing and community education campaign. Even in circumstances in which skilled midwives cannot be placed in local communities due to logistical barriers, lack of financial resources, or shortages of trained personnel, it may still be possible to reduce the consequences of obstructed labor by training, supporting, and utilizing local childbirth monitors who can at least insure that the sun does not rise twice on a laboring woman without her being sent for competent evaluation and treatment.

## Competing interests

The author is the founder of The Worldwide Fistula Fund, a not-for-profit tax-exempt 501(c)(3) public charity devoted to education, advocacy, research, and the care of women with obstetric fistulas. The preparation of this manuscript has been supported by The Worldwide Fistula Fund.

## Authors’ contributions

LLW conceived this paper, wrote and revised the manuscript, and approved the final draft submitted for publication.

## Authors’ information

LLW is Professor of Obstetric & Gynecology in the School of Medicine and Professor of Anthropology in the College of Arts and Sciences at Washington University in St. Louis. He has published widely on pelvic floor disorders, obstetric trauma, and medical anthropology. He has carried out anthropological field research in West Africa and has been instrumental in the founding of The Danja Fistula Center, a specialist hospital for women with childbirth injuries in southern Niger.

## Pre-publication history

The pre-publication history for this paper can be accessed here:

http://www.biomedcentral.com/1471-2393/12/68/prepub

## References

[B1] Neilson JP Lavender T Quenby S Wray S Obstructed labour Brit Med Bull 2003 67 191 204 10.1093/bmb/ldg018 14711764

[B2] AbouZahr C Global burden of maternal death and disability Brit Med Bull 2003 67 1 11 10.1093/bmb/ldg015 14711750

[B3] Wall LL Obstetric vesicovaginal fistula as an international public health problem Lancet 2006 368 1201 1209 10.1016/S0140-6736(06)69476-2 17011947

[B4] Yeakey MP Chipeta E Taulo F Tsui AO The lived experience of Malawian women with obstetric fistula Cult Health Soc 2009 11 499 513 10.1080/13691050902874777 19444686

[B5] Wall LL Fitsari ‘Dan Duniya: An African (Hausa) praise-song about vesico-vaginal fistulas Obstet Gynecol 2002 100 1328 1332 10.1016/S0029-7844(02)02498-5 12468180

[B6] Vangeenderhuysen C Prual A Ould el- Joud D Obstetric fistulae: incidence estimates for sub-Saharan Africa Int J Gynecol Obstet 2001 73 65 66 10.1016/S0020-7292(00)00374-X 11336724

[B7] Tebeu PM de Bernis L Doh A Sama A Rochat CH Delvauz T Risk factors for obstetric fistula in the Far North Province of Cameroon Int J Gynecol Obstet 2009 107 12 15 10.1016/j.ijgo.2009.05.019 19589525

[B8] Emmet TA Vesico-Vaginal Fistula from Parturition and Other Causes: with Cases of Recto-Vaginal Fistula 1868 William Wood, New York

[B9] Arrowsmith S Hamlin EC Wall LL Obstructed labor injury complex: Obstetric fistula formation and the multifaceted morbidity of maternal birth trauma in the developing world Obstet Gynecol Surv 1996 51 568 574 10.1097/00006254-199609000-00024 8873157

[B10] McCarthy J Maine D A framework for analyzing the determinants of maternal mortality Stud Fam Plann 1992 23 23 33 10.2307/1966825 1557792

[B11] Tsui AO Creanga AA Ahmed S The role of delayed childbearing in the prevention of obstetric fistulas Int J Gynecol Obstet 2007 99 Suppl 1 S98 S107 10.1016/j.ijgo.2007.06.024 17868676

[B12] Fortney JA The importance of family planning in reducing maternal mortality Stud Fam Plann 1987 18 109 114 10.2307/1966702 3590265

[B13] Fortney JA Antenatal risk screening and scoring: A new look Int J Gynecol Obstet 1995 50 Suppl 2 S53 S58 10.1016/0020-7292(95)02487-W 29645155

[B14] Yuster EA Rethinking the role of the risk approach and antenatal care in maternal mortality reduction Int J Gynecol Obstet 1995 50 Suppl 2 S59 S61 10.1016/0020-7292(95)02488-X 29645144

[B15] Prual A Toure A Huguet D Laurent Y The quality of risk factor screening during antenatal consultations in Niger Health Policy Plan 2000 15 11 16 10.1093/heapol/15.1.11 10731230

[B16] Dujardin B Clarysse G Mentens H De Schampheleire I Kulker R How accurate is maternal height measurement in Africa? Int J Gynecol Obstet 1993 41 139 145 10.1016/0020-7292(93)90696-T 8099029

[B17] Dujardin B Van Cutsem R Lamrechts T The value of maternal height as a risk factor of dystocia: a meta-analysis Trop Med Int Health 1996 1 510 521 10.1046/j.1365-3156.1996.d01-83.x 8765460

[B18] Moller B Lindmark G Short stature: an obstetric risk factor? A comparison of two villages in Tanzania Acta Obstet Gynecol Scand 1997 76 394 397 10.3109/00016349709047817 9197438

[B19] Zaretsky MV Alexander JM McIntire DD Hatab MR Twickler DM Leveno KJ Magnetic resonance imaging pelvimetery and the prediction of labor dystocia Obstet Gynecol 2005 106 919 926 10.1097/01.AOG.0000182575.81843.e7 16260507

[B20] Awonuga AO Merhi Z Awonuga MT Samuels TA Waller J Pring D Anthropometric measurements in the diagnosis of pelvic size: an analysis of maternal height and shoe size and computed tomography pelvimetric data Arch Gynecol Obstet 2007 276 523 528 10.1007/s00404-007-0370-0 17458554

[B21] Kasongo Project Team Antenatal screening for fetopelvic dystocias: A cost-effectiveness approach to the choice of simple indicators for use by auxiliary personnel J Trop Med Hyg 1984 87 173 183 6441000

[B22] Ronsmans C Etard JF Walraven G Hoj L Dumont A de Bernis L Kodio B Maternal mortality and access to obstetric services in West Africa Trop Med Int Health 2003 8 940 948 10.1046/j.1365-3156.2003.01111.x 14516306

[B23] Paxton A Maine D Freedman L Fry D Lobis S The evidence for emergency obstetric care Int J Gynecol Obstet 2005 88 181 191 10.1016/j.ijgo.2004.11.026 15694106

[B24] Thaddeus S Maine D Too far to walk: Maternal mortality in context Soc Sci Med 1994 38 1091 1110 10.1016/0277-9536(94)90226-7 8042057

[B25] Gabrysch S Campbell OMR Still too far to walk: Literature review of the determinants of delivery service use BMC Pregnancy Childbirth 2009 9 34 10.1186/1471-2393-9-34 19671156 PMC2744662

[B26] Bartlett LA Mawji S Whitehead S Crouse C Dalil S Ionete D Salama P the Afghan Maternal Mortality Study Team Where giving birth is a forecast of death: maternal mortality in four districts of Afghanistan, 1999–2002 Lancet 2005 365 864 870 10.1016/S0140-6736(05)71044-8 15752530

[B27] Gabrysch S Cousens S Cox J Campbell OMR The influence of distance and level of care on deliver place in Rural Zambia: A study of linked national data in a geographic information system PLoS Med 2011 8 1 e1000394 10.1371/journal.pmed.1000394 21283606 PMC3026699

[B28] Greenwood AM Greenwood BM Bradley AK Williams K Shenton FC Tulloch S Byas P Oldfield FSJ A prospective survey of the outcome of pregnancy in a rural area of the Gambia Bull World Health Org 1987 65 635 643 3501343 PMC2491067

[B29] Essien E Ifenne D Sabitu K Musa A Alti-Mu’azu M Adidu V Golji N Mukaddas M Community loan funds and transport services for obstetric emergencies in northern Nigeria Int J Gynecol Obstet 1997 59 Suppl 2 S237 S244 10.1016/s0020-7292(97)00171-9 9389637

[B30] Shehu D Ikeh AT Kuna MT Mobilizing transport for obstetric emergencies in northwestern Nigeria Int J Gynecol Obstet 1997 59 Suppl 2 S173 S180 10.1016/s0020-7292(97)00163-x 9389629

[B31] Samai O Sengeh Facilitating emergency obstetric care through transportation and communication, Bo, Sierra Leone Int J Gynecol Obstet 1997 59 Suppl 2 S157 S164 10.1016/s0020-7292(97)00161-6 9389627

[B32] Danel I Maternal Mortality Reduction, Honduras, 1990–1997: A Case Study 1998 The World Bank, Washington, DC

[B33] Pathmanathan I Liljestrand J Martins JM, Rajapaksa LC, Lissner C, de Silva A, Selvaraju S, Singh PJ: Investing in maternal health: Learning from Malaysia and Sri Lanka 2003 The World Bank, Washington, DC

[B34] Sorenson BL Elsass P Nielson BB Massawe S Nyakina J Rasch V Substandard emergency obstetric care: A confidential enquiry into maternal deaths at a regional hospital in Tanzania Trop Med Int Health 2010 5 894 900 10.1111/j.1365-3156.2010.02554.x 20545917

[B35] Sundari TK The untold story: How the health care systems in developing countries contribute to maternal mortality Int J Health Serv 1992 22 513 528 10.2190/91YH-A52T-AFBB-1LEA 1644513

[B36] Gohou V Ronsmans C Kacou L Yaou K Bohoussou KM Houphouet B Bosso P Diarra-Nama AJ Bacci A Filippi V Responsiveness to life-threatening obstetric emergencies in two hospitals in Abijan, Cote d’Ivoire Trop Med Int Health 2004 9 406 415 10.1111/j.1365-3156.2004.01204.x 14996371

[B37] Kilpatrick SJ Crabtree KE Kemp A Geller S Preventability of maternal deaths: Comparison between Zambian and American referral hospitals Obstet Gynecol 2002 100 321 326 10.1016/S0029-7844(02)02065-3 12151157

[B38] Lewis G Reviewing maternal deaths to make pregnancy safer Best Pract Res Clin Obstet Gynaecol 2008 22 447 463 10.1016/j.bpobgyn.2007.10.007 18337177

[B39] Okon P Byamugisha J Mirembe F Byaruhanga R Bergstrom S Audit of severe maternal morbidity in Uganda: Implications for quality of obstetric care Acta Obstet Gynecol 2006 85 797 804 10.1080/00016340600593331 16817076

[B40] Chamberlain J McDonagh R Lalonde A Arulkumaran S The role of professional associations in reducing maternal mortality worldwide Int J Gynecol Obstet 2003 83 94 102 10.1016/S0020-7292(03)00185-1 14511883

[B41] Klufio CA Kwawukume EY Danso KA Sciarra JJ Johnson T Ghana postgraduate obstetrics/gynecology collaborative residency training program: Success story and model for Africa Am J Obstet Gynecol 2003 189 692 696.35 10.1067/S0002-9378(03)00882-2 14526295

[B42] Kongnyuy EJ Mlava G van den Broek N A criterion based audit of the management of obstructed labour in Malawi Arch Gynecol Obstet 2009 279 649 54 10.1007/s00404-008-0786-1 18779971

[B43] Shiffman J Generating political priority for maternal mortality reduction in 5 developing countries Am J Pub Health 2007 97 796 803 10.2105/AJPH.2006.095455 17395848 PMC1854881

[B44] Shifffman J Smith S Generation of political priority for global health initiatives: a framework and case study of maternal mortality Lancet 2007 370 1370 1379 10.1016/S0140-6736(07)61579-7 17933652

[B45] de Bono E Serious Creativity: Using the Power of Lateral Thinking to Create New Ideas 1992 HarperBusiness, New York 58

[B46] Korell AN Argenta PA Strathy JH Prolonged obstructed labor causing a severe obstetric fistula: A case report J Reprod Med 2007 52 555 556 17694983

[B47] Allen AM Lakin T Shobeiri SA Nihira M Transmural vaginal-to-bladder injury from an obstructed labor pattern Obstet Gynecol 2011 117 468 470 10.1097/AOG.0b013e3181f9b749 21252792

[B48] Wall LL Karshima JA Kirschner C Arrowsmith SD The obstetric vesicovaginal fistula: Characteristics of 899 patients from Jos, Nigeria Am J Obstet Gynecol 2004 190 1011 1019 10.1016/j.ajog.2004.02.007 15118632

[B49] Muleta M Rasmussen S Kiserud T Obstetric fistula in 14,928 Ethiopian women Acta Obstet Gynecol 2010 89 945 951 10.3109/00016341003801698 20397760

[B50] Lewis A Kaufman MR Wolter CE Phillips SE Maggi D Condry L Dmochowski RR Smith JA Genitourinary fistula experience in Sierra Leone: Review of 505 cases J Urol 2009 181 1725 1731 10.1016/j.juro.2008.11.106 19230926

[B51] Hilton P Ward A Epidemiological and surgical aspects of urogenital fistulae: A review of 25 years’ experience in southeast Nigeria Int Urogynecol J 1998 9 189 194 10.1007/BF01901602 9795822

[B52] Sjoveian S Vangen S Mukwege D Onsrud Surgical outcome of obstetric fistula: a retrospective analysis of 595 patients Acta Obstet Gynecol Scand 2011 90 753 760 10.1111/j.1600-0412.2011.01162.x 21542810

[B53] Janzen J The Quest for Therapy: Medical Pluralism in Lower Zaire 1982 University of California Press, Berkeley

[B54] Sargent CF The Cultural Context of Therapeutic Choice: Obstetrical Care Decision Among the Bariba of Benin 1982 D. Reidel, Boston

[B55] World Health Organization Educational Material for Teachers of Midwifery: Midwifery Education Modules: Managing Prolonged and Obstructed Labour 2008 2 World Health Organization, Geneva

[B56] Graham EJ Bell JS Bullough CHW Can skilled attendance at delivery reduce maternal mortalilty in developing countries? Stud Health Serv Org Policy 2001 17 97 129

[B57] Scott S Ronsmans C The relationship between birth with a health professional and maternal mortality in observational studies: a review of the literature Trop Med Int Health 2009 14 1523 1533 10.1111/j.1365-3156.2009.02402.x 19793070

[B58] Prevention of Maternal Mortality Network Barriers to treatment of obstetric emergencies in rural communities of West Africa Stud Fam Plann 1992 23 279 291 1475796

[B59] Douglas M Wildavsky A Risk and Culture: An Essay on the Selection of Technical and Environmental Dangers 1982 University of California Press, Los Angeles

[B60] Douglas M Risk and Blame: Essays in Cultural Theory 1992 Routledge, New York

[B61] Obermeyer CM Risk, uncertainty, and agency: Culture and safe motherhood in Morocco Med Anthro 2000 19 173 201 10.1080/01459740.2000.9966175 11307571

[B62] McCourt Childbirth, Midwifery and Concepts of Time 2009 Berghahn Books, New York

[B63] Schauberger CW False labor Obstet Gynecol 1986 68 770 772 3785787

[B64] Anonymous World Health Organization partograph in management of labour Lancet 1994 343 1399 1404 7910888

[B65] Mathai M The partograph for the prevention of obstructed labor Clin Obstet Gynecol 2009 52 256 269 10.1097/GRF.0b013e3181a4f163 19407533

[B66] Caughey AB Is there an upper time limit for the management of the second stage of labor? Am J Obstet Gynecol 2009 201 337 338 10.1016/j.ajog.2009.08.001 19788964

[B67] Davis-Floyd RE Sargent CF (Editors): Childbirth and Authoritative Knowledge: Cross-cultural Perspectives 1997 University of California Press, Los Angeles

[B68] Adetunji JA Church-based obstetric care in a Yoruba community, Nigeria Soc Sci Med 1992 35 1171 1178 10.1016/0277-9536(92)90229-J 1439935

[B69] Udoma EJ Asuquo EEJ Ekott MI Maternal mortality from obstructed labor in south-eastern Nigeria: the role of spiritual churches Int J Gynecol Obstet 1999 67 103 105 10.1016/S0020-7292(99)00098-3 10636054

[B70] Wall LL Hausa Medicine: Illness and Well-being in a West African Culture 1988 Duke University Press, Durham, NC

[B71] Omorodion FI The socio-cultural context of health behavior among Esan communities, Edo State, Nigeria Health Transit Rev 1993 3 125 136 10146569

[B72] Wilson M Rituals of Kinship Among the Nyakyusa 1957 Oxford University Press for the International African Institute, London

[B73] Allen DR Managing Motherhood, Managing Risk: Fertility and Danger in West Central Tanzania 2002 University of Michigan Press, Ann Arbor

[B74] Chapman RR Family Secrets: Risking Reproduction in Central Mozambique; Nashville 2010 Vanderbilt University Press, TN

[B75] Berry NS Unsafe Motherhood: Mayan Maternal Mortality and Subjectivity in Post-War Guatemala 2010 Berghahn Books, New York

[B76] Anderson BA Anderson EN Franklin T Dzib-Xihum de Cen A Pathways of decision making among Yucatan Mayan traditional birth attendants J Midwifery Womens Health 2004 49 312 319 10.1016/j.jmwh.2004.03.008 15236711

[B77] Ityavyar DA A traditional midwife practice, Sokoto State, Nigeria Soc Sci Med 1984 18 6 497 501 10.1016/0277-9536(84)90007-8 6710190

[B78] Kamatenesi-Mugisha M Oryem-Origa H Medicinal plants used to induce labour during childbirth in western Uganda J Ethnopharmacol 2007 109 1 9 10.1016/j.jep.2006.06.011 16901666

[B79] Dujardin B Boutsen M De Schampheleire I Kulker R Manshande JP Bailey J Wollast E Buekens P Oxytocics in developing countries Int J Gynecol Obstet 1995 50 243 251 10.1016/0020-7292(95)02389-T 8543106

[B80] Sharan M Strobino D Ahmed S Intrapartum oxytocin use for labor acceleration in rural India Int J Gynecol Obstet 2005 90 251 257 10.1016/j.ijgo.2005.05.008 16023648

[B81] Tahzib F Vesicovaginal fistula in Nigerian children. Lancet 1985 2 1291 1293 10.1016/s0140-6736(85)91567-3 2866348

[B82] Tukur J Jido TA Uzoho CC The contribution of gishiri cut to vesicovaginal fistula in Birnin Kudu, northern Nigeria Afr J Urol 2006 12 121 125

[B83] Okojie CEE Gender inequalities of health in the Third World Soc Sci Med 1994 39 9 1237 1247 10.1016/0277-9536(94)90356-5 7801161

[B84] Doyal L Gender equity in health: debates and dilemmas Soc Sci Med 2000 51 931 939 10.1016/S0277-9536(00)00072-1 10972436

[B85] Murphy EM Being born female is dangerous for your health Am Psychol 2003 58 205 210 12772426 10.1037/0003-066x.58.3.205

[B86] Riyami A Afifi M Mabry RM Women’s autonomy, education and employment in Oman and their influence on contraceptive use Reprod Health Matters 2004 12 23 144 154 10.1016/S0968-8080(04)23113-5 15242223

[B87] Upadhyay UD Hindin MJ Do higher status and more autonomous women have longer birth intervals? Results from Cebu, Philippines Soc Sci Med 2005 60 2641 2655 10.1016/j.socscimed.2004.11.032 15814188

[B88] Duze MC Mohammed IZ Male knowledge, attitudes and family planning practices in northern Nigeria Afr J Reprod Health 2006 10 53 65 10.2307/30032471 17518131

[B89] Audu B Yahya S Geidam A Abdussalam H Takai I Kyari O Polygamy and the use of contraceptives Int J Obstet Gynecol 2008 101 88 92 10.1016/j.ijgo.2007.09.036 18082747

[B90] Evans-Pritchard EE An Alternative Term for "Bride-Price." Man 1931 31 36 39

[B91] Goody J Tambiah SJ: Bridewealth and Dowry 1973 Cambridge University Press, Cambridge

[B92] Wall LL Dead mothers and injured wives: The social context of maternal morbidityand mortality among the Hausa of northern Nigeria Stud Fam Plann 1998 29 341 359 10.2307/172248 9919629

[B93] Delaney C The Seeds and the Soil: Gender and Cosmology in Turkish Village Society 1991 University of California Press, Berkeley

[B94] Papanek H Separate worlds and symbolic shelter Comp Stud Soc History 1973 15 289 325 10.1017/S001041750000712X

[B95] VerEecke C “It is better to die than to be shamed” – Cultural and moral dimensions of women’s trading in an Islamic Nigerian society Antrhopos 1993 88 403 417

[B96] Callaway BJ Ambiguous consequences of the socialization and seclusion of Hausa women J Mod Afr Studies 1984 22 429 450 10.1017/S0022278X00055117

[B97] Solivetti LM Marriage and divorce in a Hausa community: A sociological model Africa 1994 64 252 271 10.2307/1160983 12320089

[B98] Hill P Hidden trade in Hausaland Man, n.s 1969 4 392 409

[B99] Pittin R Women, work and ideology in Nigeria Rev Afr Political Econ 1991 52 38 52

[B100] McAlister C Baskett TF Female education and maternal mortality: A worldwide survey J Obstet Gynecol Canada 2006 28 983 990 10.1016/S1701-2163(16)32294-0 17169224

[B101] Harrison K The importance of the educated healthy woman in Africa Lancet 1997 349 644 647 10.1016/S0140-6736(97)02192-2 9057747

[B102] Weeks A Lavender T Nazziwa E Mirembe F Personal account of ‘near-miss’ maternal mortalities in Kampala, Uganda BJOG 2005 112 1302 1307 10.1111/j.1471-0528.2005.00703.x 16101612

[B103] Kyomuhendo GB Low use of rural maternity services in Uganda: Impact of women’s status, traditional beliefs and limited resources Reprod Health Matters 2003 11 21 16 26 10.1016/S0968-8080(03)02176-1 12800700

[B104] Trevitt L Attitudes and customs in childbirth amongst Hausa women in Zaria City Savannah 1973 2 223 226

[B105] De Muylder X Caesarean section morbidity at district level in Zimbabwe J Trop Med Hyg 1989 92 89 92 2709481

[B106] Kwawukume EY Caesarean section in developing countries Best Pract Res Clinical Obstet and Gynaecol 2001 15 165 178 10.1053/beog.2000.0155 11359321

[B107] Oladapo OT Lamina MA Sule-Odu AO Maternal morbidity and mortality associated with elective caesarean delivery at a university hospital in Nigeria Austral NZ J Obstet Gynaecol 2007 47 110 114 10.1111/j.1479-828X.2007.00695.x 17355299

[B108] De Muylder X de Waals P Poor acceptance of caesarean section in Zimbabwe Trop Geograph Med 1989 41 230 233 2595801

[B109] Chigbu CO Ilobachie GC The burden of caesarean section refusal in a developing country setting BJOG 2007 114 1261 1263 10.1111/j.1471-0528.2007.01440.x 17877678

[B110] Onah HE Formal education does not improve the acceptance of cesarean section among pregnant Nigerian women Int J Gynecol Obstet 2002 76 321 323 10.1016/S0020-7292(01)00578-1 11880141

[B111] Onah HE Nkwo PO Caesarean section or symphysiotomy for obstructed labour for developing countries? Need to ascertain women’s preferences J Obstet Gynaecol 2003 23 594 595 10.1080/01443610310001604312 14617456

[B112] Parkhurst JO Rahman SA Life saving or money wasting? Perceptions of caesarean sections among users of services in rural Bangladesh Health Policy 2007 80 392 401 10.1016/j.healthpol.2006.03.015 16698113

[B113] Kowalewski M Jahn A Kimatta SS Why do at-risk mothers fail to reach referral level? Barriers beyond distance and cost Afr J Reprod Health 2000 4 100 109 10.2307/3583247 11000713

[B114] Asuquo EEJ Duke F Staff attitude as barrier to the utilization of University of Calabar Teaching Hospital for obstetric care Afr J Reprod Health 2000 4 69 73 10.2307/3583450

[B115] Grossman-Kendall F Filippi V De Koninck M Kanhonou L Giving birth in maternity hospitals in Benin: Testimonies of women Reprod Health Matters 2001 9 18 90 98 10.1016/S0968-8080(01)90095-3 11765405

[B116] Kongnyuy EJ Mlava G van den Broek Nynke Criteria-based audit to improve women-friendly care in maternity units in Malawi J Obstet Gynaecol Res 2009 35 483 489 10.1111/j.1447-0756.2008.00990.x 19527387

[B117] Phillips D Medical professional dominance and client dissatisfaction: A study of doctor-patient interaction and reported dissatisfaction with medical care among female patients at four hospitals in Trinidad and Tobago Soc Sci Med 1996 42 1419 1425 10.1016/0277-9536(95)00290-1 8735898

[B118] Miller S Cordero M Coleman AL Figueroa J Brito-Anderson S Dabagh R Calderon V Caceres V Fernandez AJ Nunez M Quality of care in institutionalized deliveries: The paradox of the Dominican Republic Int J Gynecol Obstet 2003 82 89 103 10.1016/S0020-7292(03)00148-6 12834953

[B119] Mayi-Tsonga S Oksana L Ndombi I Diallo T de Sousa MH Faoundes A Delay in the provision of adequate care to women who died from abortion-related complications in the principal maternity hospital of Gabon Reprod Health Matters 2009 17 34 65 70 10.1016/S0968-8080(09)34465-1 19962639

[B120] Jaffre Y Prual A Midwives in Niger: An uncomfortable position between social behaviours and health care constraints Soc Sci Med 1994 38 1069 1073 10.1016/0277-9536(94)90224-0 8042055

[B121] Ozumba BC Anya SE Maternal deaths associated with cesarean section in Enugu, Nigeria Int J Gynecol Obstet 2002 76 307 309 10.1016/S0020-7292(01)00501-X 11880136

[B122] Short SE Zhang F Use of maternal health services in rural China Popul Stud 2004 58 3 19 10.1080/0032472032000175446 15204259

[B123] Abel-Smith B Rawal P Can the poor afford ‘free’ health services? A case study of Tanzania Health Policy Plan 1992 7 329 342 10.1093/heapol/7.4.329

[B124] Kowalewski M Mujinja P John A Can mothers afford maternal health care costs? User costs of maternity services in rural Tanzania Afr J Reprod Health 2002 6 65 73 10.2307/3583147 12476730

[B125] Su TT Kouyate B Flessa S Catastrophic household expenditure for health care in a low-income society: a study from Nouna District, Burkina Faso Bull World Health Org 2006 84 21 27 16501711 10.2471/blt.05.023739 PMC2626518

[B126] Borghi J Hanson K Acquah CA Ekanmian G Filippi V Ronsmans C Brugha R Browne E Alihonou E Cost of near-miss obstetric complications for women and their families in Benin and Ghana Health Policy Plan 2003 18 383 390 10.1093/heapol/czg046 14654514

[B127] Borghi J Ensor T Neupane BD Tiwari S Financial implications of skilled attendance at delivery in Nepal Trop Med Int Health 2006 11 228 237 10.1111/j.1365-3156.2005.01546.x 16451348

[B128] Nahar S Costello A The hidden cost of ‘free’ maternity care in Dhaka, Bangladesh Health Policy Plan 1998 13 417 422 10.1093/heapol/13.4.417 10346033

[B129] Houweling TAJ Ronsmans C Campbell OMR Kunst A Huge poor-rich inequalities in maternity care: an international comparative study of maternity and child care in developing countries Bull World Health Org 2007 85 745 754 18038055 10.2471/BLT.06.038588 PMC2636501

[B130] Storeng KT Baggaley RF Ganaba R Ouattara F Akoum MS Filippi V Paying the price: The cost and consequences of emergency obstetric care in Burkina Faso Soc Sci Med 2008 66 545 557 10.1016/j.socscimed.2007.10.001 18061325

[B131] Borghi J Sabina N Blum LS Hoque HE Ronsmans C Household costs of healthcare during pregnancy, delivery, and the postpartum period: A case study from Matlab, Bangladesh J Health Pop Nutrition 2006 24 446 455 17591341 PMC3001148

[B132] Say L Rainse R A systematic review of inequalities in the use of maternal health care in developing countries: Examining the scale of the problem and the importance of context Bull World Health Org 2007 85 812 819 18038064 10.2471/BLT.06.035659 PMC2636485

[B133] Afsana K The tremendous cost of seeking hospital obstetric care in Bangladesh Reprod Health Matters 2004 12 24 171 180 10.1016/S0968-8080(04)24142-8 15626207

[B134] Mbugua JK Bloom GH Segall MM Impact of user charges on vulnerable groups: The case of the Kibwezi in rural Kenya Soc Sci Med 1995 41 829 835 10.1016/0277-9536(94)00400-N 8571154

[B135] Nanda P Gender dimensions of user fees: Implications for women’s utilization of health care Reprod Health Matters 2002 10 20 127 134 10.1016/S0968-8080(02)00083-6 12557649

[B136] Xu K Evans DB Kadama P Nabyonga J Ogwal PO Nabukhonzo P Aguilar AM Understanding the impact of eliminating user fees: Utilization and catastrophic health expenditures in Uganda Soc Sci Med 2006 62 866 876 10.1016/j.socscimed.2005.07.004 16139936

[B137] Kruk ME Mbaruku G Rockers PC Galea S User fee exemptions are not enough: out-of-pocket payments for ‘free’ delivery services in rural Tanzania Trop Med Int Health 2008 13 1442 1451 10.1111/j.1365-3156.2008.02173.x 18983268

[B138] Lewis M Informal payments and the financing of health care in developing and transition countries Heal Aff 2007 26 984 997 10.1377/hlthaff.26.4.984 17630441

[B139] Khan A Aman S Costs of vaginal delivery and Caesarean section at a tertiary level public hospital in Islamabad, Pakistan BMC Pregnancy Childbirth 2010 10 10.1186/1471-2393-10-2 PMC2826286 20085662

[B140] McIntyre D Thiede M Dahlgren G Whitehead M What are the economic consequences for households of illness and paying for health care in low- and middle-income country contexts? Soc Sci Med 2006 62 858 865 10.1016/j.socscimed.2005.07.001 16099574

[B141] Ronsmans C Holtz S Stanton C Socioeconomic differentials in caesarean rates in developing countries: a retrospective analysis Lancet 2006 368 1516 1523 10.1016/S0140-6736(06)69639-6 17071285

[B142] Russell S Ability to pay for health care: concepts and evidence Health Policy Plan 1996 11 219 237 10.1093/heapol/11.3.219 10160370

[B143] Gilson L Trust and health care as a social institution Soc Sci Med 2003 67 1452 1468 10.1016/s0277-9536(02)00142-9 12614697

[B144] Shore D A (Editor): The Trust Crisis in Healthcare: Causes, Consequences, and Cures 2006 Oxford University Press, New York

[B145] Gilson L Palmer N Schneider H Trust and health worker performance: exploring a conceptual framework using South African evidence Soc Sci Med 2005 61 1418 1429 10.1016/j.socscimed.2004.11.062 16005777

[B146] Harrison KA Child-bearing, health and social priorities: A survey of 22,774 consecutive hospital births in Zaria, northern Nigeria Brit J Obstet Gynaecol 1985 92 Suppl 5 1 119 4052352

[B147] Ekele BA Audu LR Muyibi S Uterine rupture in Sokoto, northern Nigeria: Are we winning? Afr J Med Med Sci 2000 29 191 193 11713987

[B148] Diab AE Uterine ruptures in Yemen Saudi Med J 2005 26 264 269 15770303

[B149] El Jould DO Prual A Vangeenderhuysen C Bouvier-Colle MH the MOMA Group Epidemiological features of uterine rupture in West Africa (MOMA Study) Paed Perinat Epidemiol 2002 16 108 114 10.1046/j.1365-3016.2002.00414.x 12060311

[B150] Chuni N Analysis of uterine rupture in a tertiary center in Eastern Nepal: Lessons for obstetric care J Obstet Gynaecol Res 2006 32 574 759 10.1111/j.1447-0756.2006.00461.x 17100819

[B151] Mishra SK Morris N Uprety DK Uterine rupture: Preventable obstetric tragedies? Austral NZ J Obstet Gynaecol 2006 46 541 545 10.1111/j.1479-828X.2006.00665.x 17116062

